# Factors that influence health literacy in patients with coronary artery disease *

**DOI:** 10.1590/1518-8345.6211.3879

**Published:** 2023-03-27

**Authors:** Ana Caroline da Costa, Ana Paula da Conceição, Howard Karl Butcher, Rita de Cassia Gengo e Silva Butcher

**Affiliations:** 1 Faculdade Wenceslau Braz, Departamento de Enfermagem, Itajubá, MG, Brasil; 2 Universidade de São Paulo, Escola de Enfermagem, São Paulo, SP, Brasil; 3 Instituto Dante Pazzanese de Cardiologia, Departamento de Enfermagem, São Paulo, SP, Brasil; 4 Florida Atlantic University, Christine E. Lynn College of Nursing, Boca Raton, Florida, Estados Unidos da América

**Keywords:** Health Literacy, Coronary Artery Disease, Nursing, Health Education, Nursing Care, Socioeconomic Factors, Alfabetización en Salud, Enfermedad Arterial Coronaria, Enfermería, Educación en Salud, Atención de Enfermería, Factores Socioeconómicos, Letramento em Saúde, Doença da Artéria Coronariana, Enfermagem, Educação em Saúde, Cuidados de Enfermagem, Fatores Socioeconômicos

## Abstract

**Objective:**

to investigate the factors that exert an influence on health literacy in patients with coronary artery disease.

**Methods:**

a crosssectional study, including 122 patients with coronary diseases (60.7% male; 62.07 ± 8.8 years old). Health literacy and specific knowledge about the disease were evaluated through interviews with the participants by means of the Short Test of Functional Health Literacy in Adults and the Short version of the coronary artery disease education questionnaire. The data were described by means of central tendency measures and frequencies. The factors that exert an influence on health literacy were determined by means of a linear regression model. The significance level adopted was 5%. The study was approved by the Research Ethics Committee.

**Results:**

age and arterial hypertension presented an inverse and significant relationship with health literacy. On the other hand, higher schooling levels and having a job were associated with better scores in the health literacy instrument. Specific knowledge about the disease did not exert any influence on health literacy. The variables included in the regression model explained 55.3% of inadequate literacy.

**Conclusion:**

this study, knowledge about the disease exerts no influence on health literacy: however, the professionals should consider the sociodemographic and clinical factors to plan the interventions.

## Abstract (es)

**Objetivo:**

investigar los factores que influyen en la alfabetización en salud de los pacientes con enfermedad arterial coronaria.

**Método:**

estudio transversal, que incluyó 122 pacientes con enfermedades coronarias (60,7% del sexo masculino; 62,07±8,8 años); se evaluó la alfabetización en salud y el conocimiento específico sobre la enfermedad mediante entrevistas con los participantes, utilizando el Short Test of Functional Health Literacy in Adults e Short version of the coronary artery disease education questionnaire. Los datos fueron descritos por medidas de tendencia central y frecuencias. Los factores que influyen en la alfabetización en salud se determinaron mediante un modelo de regresión lineal. El nivel de significación adoptado fue del 5%. El estudio fue aprobado por el Comité de Ética e Investigación.

**Resultados:**

la edad y la hipertensión mostraron una relación inversa y significativa con la alfabetización en salud. Por otro lado, un mayor nivel educativo y tener empleo se asociaron con puntajes más altos en el instrumento de alfabetización en salud. El conocimiento específico sobre la enfermedad no influyó en la alfabetización en salud. Las variables del modelo de regresión explicaron el 55,3% de alfabetización inadecuada.

**Conclusión:**

en el presente estudio, se concluyó que el conocimiento sobre la enfermedad no influye en la alfabetización en salud, pero los profesionales deben considerar los factores sociodemográficos y clínicos para planificar las intervenciones.

## Abstract (pt)

**Objetivo:**

investigar os fatores que influenciam o letramento em saúde em pacientes com doença arterial coronariana.

**Método:**

estudo transversal, incluindo 122 pacientes com coronariopatias (60,7% do sexo masculino; 62,07±8,8 anos); letramento em saúde e conhecimento específico da doença foram avaliados por meio de entrevista com os participantes, pelo Short Test of Functional Health Literacy in Adults e Short version of the coronary artery disease education questionnaire. Os dados foram descritos por medidas de tendência central e frequências. Fatores que influenciam o letramento em saúde foram determinados por modelo de regressão linear. O nível de significância adotado foi de 5%. O estudo foi aprovado pelo Comitê de Ética e Pesquisa.

**Resultados:**

idade e hipertensão apresentaram uma relação inversa e significativa com letramento em saúde. Por outro lado, maior escolaridade e estar empregado associaram-se com maiores pontuações no instrumento de letramento em saúde. O conhecimento específico da doença não influenciou o letramento em saúde. As variáveis do modelo de regressão explicaram 55,3% do letramento inadequado.

**Conclusão:**

no presente estudo o conhecimento sobre a doença não influência o letramento em saúde, mas os profissionais devem considerar os fatores sociodemográficos e clínicos para planejar as intervenções.

**Highlights**

(1) Specific knowledge about the disease does not exert any influence on HL in patients with CAD.

(2) HL is positively influenced by higher schooling levels and better work status.

(3) HL is negatively influenced by age and arterial hypertension.

(4) The factors that exert an influence on HL should be included in educational interventions.

## Introduction

Complexity of the varied information and treatment options is acknowledged as a barrier for a successful implementation of health interventions. Using unnecessarily complicated language or inaccurate translations in educational materials, or other education sources for patients generally leads to poorly informed decision-making and decreased participation of the patients in interventions with potential benefits for health. Such complexity exerts a negative impact on health results, especially among patients with low health literacy (HL) ^(1- 2)^ levels. Health literacy is defined as a person’s ability to acquire, process and understand all the health-related information required to make suitable decisions and achieve positive health results. It involves acquiring specific skills to perform activities of daily living related to health and tasks, as well as making decisions to improve health outcomes ^(3- 4)^ . 

The prevalence of low HL is relatively high at the global level, particularly among people with low socioeconomic status ^(5)^ . A number of research studies have evidenced that low HL is an independent health determinant and that it is associated with worse health results, such as increased hospitalizations, use of emergency services, low adherence to medication regimes and higher mortality rates. In addition, patients with low HL have more difficulty understanding health information, less knowledge about their disease and less support to discuss health problems, in addition to feeling less comfortable communicating with health professionals and discouraged or even embarrassed, to ask questions in order to clarify the information they received ^(5- 6)^ . On the other hand, the literature shows higher HL levels are associated with better health self-management indicators such as adherence to healthy lifestyles and lower rates of obesity, smoking and hospital readmissions ^(7- 8)^ . 

Inadequate HL is acknowledged as a barrier for health maintenance and prevention of coronary artery disease (CAD) and is associated with non-adoption of self-care behaviors for management of the disease ^(9- 11)^ . In Brazil, a developing country with profound social inequalities and weaknesses in its health and education systems, studies on HL are still scarce, specifically in patients with CAD. To the present date, no national studies analyzing HL in this group of patients have been found, however, data from other groups of patients show that the HL level in the country is low or limited ^(12- 15)^ . 

Specifically in patients with CAD, the literature evidences that the prevalence of inadequate HL is considered high, with a frequency of up to 74.5% and that it has been associated with unfavorable outcomes for health, such as less knowledge about the disease and non-adherence to the treatment ^(9- 10)^ . A systematic review that synthesized the literature in relation to HL in people with CAD showed knowledge about the disease, age, schooling, socioeconomic disadvantaged status, non-white ethnicity and comorbidities are associated with low HL ^(9)^ . 

According to the conceptual model on HL, widely discussed in the literature, text comprehension, numbering, vocabulary and prior knowledge are necessary resources to effectively deal with health information ^(4)^ . However, the influence of specific knowledge about CAD on HL is not fully understood. Although some studies have shown the relationship between HL and specific knowledge about the disease in different groups of patients ^(11, 16- 17)^ , other surveys that included patients with chronic and infectious diseases failed to demonstrate that specific knowledge about the disease influences HL ^(18- 21)^ . A meta-analysis that included more than 18,000 participants with diabetes showed that higher HL levels were associated with higher levels of knowledge about the disease (r=0.308, p<0.001) and with better clinical outcomes, such as lower levels of glycated hemoglobin (HbA1C) (r =-0.048, p = 0.027) ^(18)^ . 

Genuine patient-centered care, especially in patients with CAD, will only be attained if high HL levels are taken into account ^(22)^ . Knowing the factors associated with HL will increase success: in the implementation of health interventions in CAD management, directing and adapting the communication between health professionals and patients and planning of educational interventions. Thus, the current study investigated the factors that exert an influence on health literacy in patients with CAD. 

## Method

### Type of study

This is an observational and cross-sectional study with a quantitative approach that is part of a main project aimed at analyzing the association between self-care and HL in patients with CAD.

This study followed The Strengthening the Reporting of Observational Studies in Epidemiology (STROBE) guideline ^(23)^ . 

### Data collection setting

Data collection was performed in the outpatient service for coronary diseases of a tertiary-level hospital in the city of São Paulo, Brazil. Choice of hospital was due to the fact that it is a reference in care and research in the Cardiology area in Brazil, in addition to being renowned worldwide for its excellence. In 2018, the hospital had 376 beds and served nearly 178,927 outpatients, among which 13,973 were registered in the Coronary diseases outpatient service.

### Period

The data collection period was from August to October 2019.

### Population and sample

The population consisted of patients with CAD registered in the tertiary-level Coronary diseases outpatient service, a reference in Cardiology from the city of São Paulo, Brazil.

For the main study that aimed at “Analyzing the association between self-care ability, health literacy and knowledge about the disease in patients with CAD”, the minimum sample size was 84 participants, calculated by means of the G Power statistical program, version 3.1 ^(24)^ , based on an infinite population, moderate correlation (r = 0.30), 80% test power and 5% type I error. The correlation value adopted for sample size calculation allows analyzing the existence of a correlation between the variables of interest, without excessively increasing the sample size to the point of rendering the study unfeasible. 

The individuals included in the study were those aged at least 18 years old, with a medical diagnosis of CAD documented in their medical charts and who stated being able to read. The Mini-Mental State Examination (MMSE) was used as pre-screening tool to determine presence of cognitive impairment, when the score obtained by the participants was below 20. This pre-screening is necessary because impairment in cognitive functions might result in poor performance in the other assessments due to difficulties understanding the tasks. This procedure is well-adopted in the literature ^(15)^ . The participants excluded from the study were those with deficits in visual, auditory and/or verbal communication skills that precluded application of the data collection instruments. 

### Procedures for data collection

The potential study participants, that is, those aged at least 18 years old and with a medical diagnosis of CAD, were identified based on the coronary diseases outpatient service schedule of appointments. Subsequently, the main researcher herself invited the potential participants to the study. Those who agreed signed the informed consent form and answered MMSE. For the participants included in the study, individual meetings were held to apply the other data collection instruments, as described below.

### Instruments for data collection

The sociodemographic and clinical data were collected from the medical charts or through the participants’ self-reports. The data of interest for this study were sex, age, race, schooling, marital status, professional status, *per capita* income of the patients, number of medications, smoking habit, comorbidities (hypertension, diabetes and dyslipidemia) and previously-received health education. 

The HL level was assessed by means of the Short Test of Functional Health Literacy in Adults (S-TOFHLA), validated for use in Brazil ^(12)^ . Choice of this instrument was due to the fact that it assesses both reading comprehension and numbering. S-TOFHLA contains 36 reading comprehension items organized into two passages: A and B. Passage A includes information related to a gastrointestinal examination and Passage B refers to a term of rights and responsibilities of a patient admitted to a hospital. Each passage has a space (each space corresponds to an item) and, below each space, there are five or six word options, from which the participant has to select one to complete the sentence and make sense of the it. Two points are assigned to each word that is properly selected, so that the total score is 72 points. 

In turn, the numbering assessment consists in presenting four cards for the participant to interpret the information. The first card is a medication label. The second refers to how to interpret a glycaemia value. Card three deals with the date of next appointment, considering the one printed in the card. Finally, in card four, the participant has to calculate the time to take a medication. Two points are assigned to each correct answer, so that the numbering component has a total score of 28 points.

It is recommended that the reading comprehension and numbering components should be finished in seven and five minutes, respectively, therefore, application of the instrument should be timed.

The total S-TOFHLA score varies from 0 to 100 and comprises the sum of the reading comprehension (from 0 to 72) and numbering (from 0 to 28) scores. Based on the score obtained, the participants’ HL level is classified as inadequate (from 0 to 53), marginal or borderline (from 54 to 66) or adequate (from 67 to 100). The original instrument’s internal consistency was 0.68 for the four numbering items and 0.97 for the 36 items from both passages of the reading comprehension test ^(12, 25)^ . 

The patients’ knowledge about CAD was evaluated through the Short Version of the Coronary Artery Disease Education Questionnaire (CADE-Q SV), also validated for use in Brazil ^(26)^ and because it is a specific instrument for assessing knowledge about CAD. The questionnaire consists of 20 items and is organized into four knowledge areas: clinical condition, risk factors, exercise, diet and psychosocial risk. Each of the four areas has four items that are randomly arranged. The answer options are as follows: “True”, “False” and “I don’t know”. Each correct answer equals one point; therefore, the total score varies from 0 to 20. The higher the score, the better the patient’s knowledge about CAD. The Brazilian version of the instrument presented adequate evidence in terms of reproducibility and its intraclass correlation coefficient was greater than 0.70 for all the items ^(26)^ . 

### Data treatment and analysis

The data were analyzed with the aid of the R software, version 3.5.380. Absolute and relative frequencies were calculated for the categorical variables; and mean, standard deviation, median and maximum and minimum values were calculated for the quantitative ones. The difference in the participants’ distribution in relation to the HL categories was determined by means of the chi-square test. A linear regression model was used to determine the influence of the patients’ previous knowledge and sociodemographic characteristics on HL. The researchers selected the independent variables based on the theoretical framework after a comprehensive reading of studies about the factors that influence HL levels.

Normality of the regression analysis residuals was evaluated by means of Q-Q plots. Multicollinearity of the independent variables was assessed by means of the variance inflation factor. The significance level adopted in all the tests was 5%, with 95% confidence intervals.

Internal consistency of the instruments used to measure HL and knowledge about CAD in the sample of this study was calculated by means of Cronbach’s alpha ^(27)^ . 

### Ethical aspects

The study was approved by the Research Ethics Committee at the hospital and the Nursing School of the University of São Paulo, with Certificate of Presentation for Ethical Appreciation ( *Certificado de Apresentação para Apreciação* Ética, CAAE) number 12805619.5.3001.5462 and 12805619.5.0000.5392, respectively. All the participants freely signed an Informed Consent Form. 

## Results

The sociodemographic data and clinical characteristics of the sample are detailed in Table 1. The participants’ mean age was 62.1 ± 8.8 years old and the mean schooling level reported corresponded to 7.8 ± 4.2 years. Most of the patients were male, Caucasian, married, and earned incomes of less than one minimum wage. Only 26.2% were employed, 63.9% were retired and 9.8% were unemployed. The vast majority (>90%) had comorbidities such as hypertension and dyslipidemia. 

**Table 1:** Sociodemographic characteristics of participants (n=122) of the study in the assessments of health literacy and knowledge about coronary artery disease. São Paulo, Brazil, 2019

Variables	
Mean age (SD^*^)	62.1 ± 8.8
Schooling, mean (SD^*^)	7.8 ± 4.2
Male sex, n (%)	74 (60.7%)
Skin color, white, n (%)	71 (58.2%)
Marital status, married, n (%)	90 (73.8%)
Work status, active, n (%)	32 (26.2%)
*Per capita*income > 1 minimum wage^†^, yes, n (%)	86 (70.5%)
Smoker, yes, n (%)	9 (7.4%)
Hypertension, n (%)	119 (97.5%)
Dyslipidemia, n (%)	114 (93.4%)
Diabetes, n (%)	59 (48.4%)

* SD = Standard deviation;

† Minimum wage in force = R$ 998.00, Brazil, 2019

**Figure 1:**
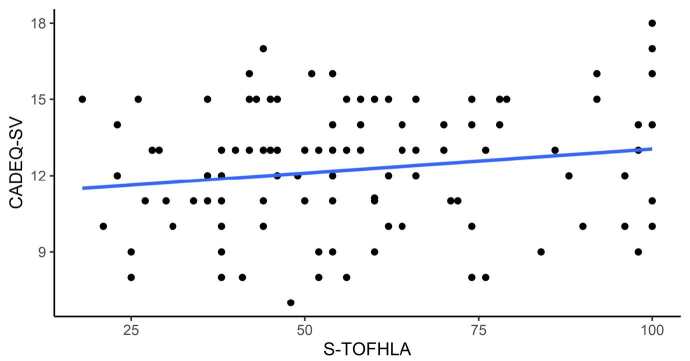
Correlation of the scores obtained by the study participants (n = 122) in the health literacy and knowledge about coronary artery disease assessments. São Paulo, Brazil, 2019

The mean total S-TOFHLA score was 60.5 ± 23.1, whereas the mean reading comprehension score was 33.6 ± 21.7 and the numbering score was 26.7 ± 2.3; 41.8% (n = 51) of the patients had inadequate HL, 35.2% (n = 43) had adequate HL and 23.0% (n = 28) had borderline HL (p = 0.035). Internal consistency of S-TOFHLA was 0.96 for reading comprehension and 0.12 for numbering. The mean CADE-Q SV score was 12.3 ± 2.5 and its internal consistency was 0.47. Figure 1 shows that there was no statistically significant correlation between the CADE-Q SV and S-TOFHLA scores. 

r* = 0.175; p ^†^ = 0.054; 95% CI ^‡^ 0.003-0.342; *r = Pearson’s correlation coefficient; ^†^p = Significance level; ^‡^CI = Confidence Interval; CADE-Q SV = Coronary Artery Disease Education Questionnaire – Short Version; S-TOFHLA = Short Test of Functional Health Literacy in Adults

Table 2 shows the linear regression model corresponding to the factors that exert an influence on HL. Each additional year of age decreased the S-TOFHLA score by 0.55 points (95% CI = -0.966; -0.125) and having hypertension decreased such score by 29.9 points in relation to those without hypertension (95% CI = -54.124; -5.696, p = 0.016). Each additional year of schooling increased the S-TOFHLA score by 2.14 points (95% CI = 1.230; 3.045, p < 0.001) and being employed increased the S-TOFHLA score by 8.56 points with respect to those who were not (95% CI = 0.422; 16.695, p = 0.039). The variables included in the regression model explain 55.3% of inadequate HL in the sample under study. 

**Table 2:** Factors that exert an influence on health literacy (n = 122), adjusted for confusion variables (arterial hypertension, diabetes *mellitus*and dyslipidemia). São Paulo, Brazil, 2019

Variables	Regression coefficient	Standard error	CI* 95% min	CI* 95% max	p value†
Male sex	4,104	3,275	-2,388	10,596	0,213
Age	-0,546	0,212	-0,966	-0,125	*0,012*
Skin color, non-white	-6,381	3,231	-12,785	0,024	0,051
Schooling	2,137	0,458	1,230	3,045	*<0,001*
Marital status, not married	5,774	3,844	-1,846	13,393	0,136
Work status (employed)	8,559	4,104	0,422	16,695	*0,039*
*Per capita*income	1,943	1,593	-1,215	5,102	0,225
Number of medications	-0,120	1,071	-2,243	2,003	0,911
Health guidance‡	3,788	3,552	-3,253	10,829	0,289
Hypertension	-29,910	12,215	-54,124	-5,696	*0,016*
Diabetes *Mellitus*	-5,686	3,707	-13,034	1,663	0,128
Dyslipidemia	8,536	7,727	-6,781	23,854	0,272
CADE-Q SV§	0,698	0,661	-0,612	2,008	0,293

* CI = Confidence Interval: Min. - Minimum Max.- Maximum;

† p = Significance level;

‡ Health guidance = It refers to any guideline or information received about health, examinations and treatments in general and not specifically about coronary artery disease;

§ CADE-Q SV = Coronary Artery Disease Education Questionnaire – Short Version

**Figure 2:**
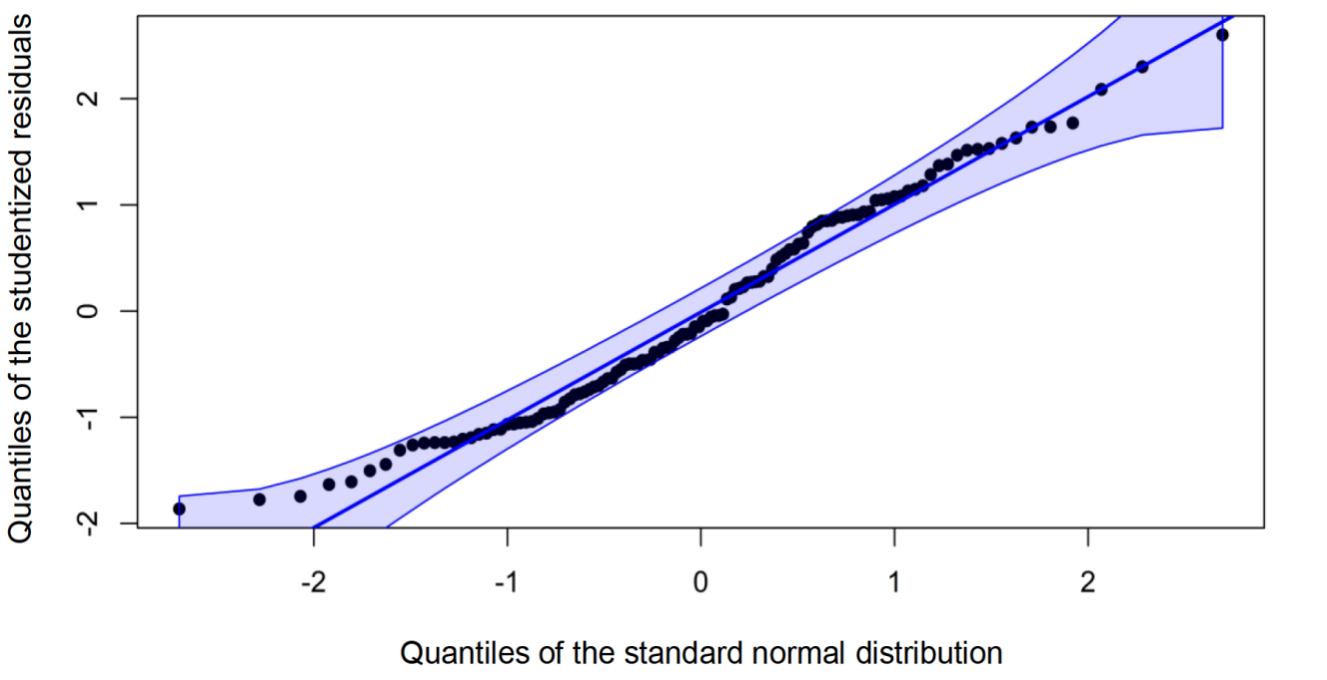
Analysis of the normality of the residuals of the variables included in the regression model (n = 122). São Paulo, Brazil, 2019

In addition, normal distribution was observed for the residuals of the variables included in the regression model by means of Q-Q graph, as shown in Figure 2. 

Table 3 shows that there was no evidence of multicollinearity among the variables included in the regression model. The highest value obtained was 1.78, which does not indicate presence of multicollinearity. 

**Table 3:** Multicollinearity analysis of the variables included in the regression model (n = 122). São Paulo, Brazil, 2019

Variables	VIF^*^
Sex	1.17
Age	1.58
Skin color	1.16
Schooling	1.69
Marital status	1.31
Work status	1.49
*Per capita* income	1.32
Number of medications	1.78
Health guidance^‡^	1.14
Hypertension	1.64
Diabetes *Mellitus*	1.57
Dyslipidemia	1.67
CADE-Q SV^§^	1.23

* VIF = Variance Inflation Factor;

‡ Health guidance = It refers to any guideline or information received on health, examinations and treatments in general and not specifically on coronary artery disease;

§ CADE-Q SV = Coronary Artery Disease Education Questionnaire – Short Version

The *a posteriori* analysis showed that 122 subjects was an adequate sample size to demonstrate that, at least one variable (predictor), significantly altered the dependent variable (HL assessed by means of the Short Test of Functional Health Literacy in Adults). Assuming a 5% type I error and 20% type II error, with 80% test power and considering that the regression model consisted of 14 predictors with a coefficient of determination (R ^2^) of 0.553, the required sample would have been 30 participants. 

## Discussion

This study analyzed the influence of specific knowledge about the disease, sociodemographic and clinical factors, including comorbidities (hypertension, diabetes and dyslipidemia and number of medications taken daily) on HL in patients with CAD. The mean S-TOFHLA score was 60.5 ± 23.1, with a statistically significant predominance of patients with inadequate HL. The frequency of inadequate HL levels in patients with CAD varies widely in the literature (from 14.3% to 74.5%) ^(9- 10)^ . However, several studies show that inadequate HL is common in patients with cardiovascular diseases in general ^(12- 15)^ , particularly in those with CAD ^(9- 10)^ . 

In this study, the factors that influenced HL were age, schooling, employment and hypertension. There was a reduction in HL with increasing age and diagnosis of hypertension, while individuals with some professional occupation and with more years of study presented higher HL levels. A systematic review also showed that the HL level was worse in unemployed people, of advanced age, with low schooling levels and multiple comorbidities ^(9)^ . 

Other authors agree that HL is a multidimensional concept influenced by personal, family, environmental and social factors. They also consider that the social factors tend to exert a strong influence not only on development of the skills required for HL, but also on the way in which patients use health information ^(4, 28- 29)^ . 

The literature shows that low HL is associated with poor blood pressure control ^(30)^ . Although there is evidence that hypertensive people with adequate HL have better adherence to the treatment ^(29, 31)^ , a study showed that the contribution of HL to explain such adherence was minimal ^(30)^ . 

In relation to age, the older adults’ low performance in tests that evaluate HL can be attributed to decline in cognition, hearing and vision, as well as to less access to educational information in the past ^(31)^ . A study showed that participants with lower S-TOFHLA scores, classified as inadequate or marginal, were significantly older, prone to be dependent on daily activities and to perform worse in cognitive domains tests, such as MMSE ^(32)^ . In another study that included a sample of 575 patients with heart failure, the authors showed that older patients were more likely to have low HL and that the health outcomes (readmissions and mortality) were worse in those aged over 65 years and with low HL. In addition to that, the authors were able to show that HL mediates the relationship between age and health results ^(33)^ . 

With regard to schooling, the findings of this study are consistent with the literature, where it is observed that individuals with lower schooling levels have lower HL scores, worse reading skills, less autonomy to seek health information in different sources and difficulty understanding and judging what is most appropriate for their own well-being ^(25, 34)^ . A cross-sectional study that included 252 patients with cardiovascular diseases showed that the lower the schooling level, the lower the HL level. The authors showed that patients without schooling had lower scores in all nine dimensions of the HL instrument used in the study (Health Literacy Questionnaire - HLQ) ^(34)^ . In a systematic review, education was considered a predictive factor for HL in patients with heart failure. The studies included in that review showed that the patients who had less than High School were more likely to have low HL levels ^(35)^ . 

Individuals with Higher Education tend to have better reading skills, more autonomy to seek health information in different sources and better ability to understand and judge what is most appropriate for their own well- being, in addition to having other positive attitudes and behaviors ^(25, 34)^ . It is possible that individuals with high schooling levels feel safer and have more clarity when communicating their needs to health professionals ^(34- 35)^ . 

In relation to employment, work status is considered as the economic inclusion degree that grants or restricts people’s access to the necessary resources ^(36)^ . Thus, being employed can be seen as an indicator of socioeconomic status. Actually, socioeconomic status does not directly affect health status. However, it was recognized as an important determinant of health-related results ^(37)^ . Those who are unemployed, retired or on sick leave and who earn low incomes and are devoid of additional health resources such as health insurance, tend to have low adherence to preventive practices and less contact with health-related information ^(37)^ . In addition, as they are not part of the workforce, they may feel disabled and vulnerable. As a result, they delegate some activities to family members, such as scheduling appointments and exams or controlling drug therapy and end up distancing themselves from the health system, thus limiting communication with professionals and, in some cases, not making decisions related to their own health ^(38)^ . Recently, a study also showed that HL is a mediator of the relationship between socioeconomic level and health status ^(37)^ . 

Regarding knowledge about the disease, CADE-Q SV has been used in few studies, which hinders comparisons. In the sample under study, the mean CADE-Q SV score was 12.3 ± 2.5 (possible range from 0 to 20). In the validation study of this instrument in Brazil, the authors found a mean CADE-Q SV score of 13.1 ^(26)^ . The findings of another research conducted with Brazilian and Canadian patients suggested that the subjects had good knowledge about the disease ^(39)^ . It is possible that socioeconomic and cultural differences may influence these findings. There is diverse evidence in the literature that lower levels of knowledge related to the disease are associated with lower schooling levels, lower incomes and advanced age ^(26)^ . In fact, among patients with CAD, although knowledge about the disease seems to be a predictor of decision-making, it is not sufficient to change health behaviors ^(40)^ . A number of authors found even though CAD patients had moderate knowledge about the disease, less than one-third of the sample was consistently involved in physical activities or underwent regular follow-up with health providers ^(40)^ . 

Our findings showed, although considered relevant by professionals, previous knowledge about the disease was not related to HL. The assumption that knowledge is necessary for HL, not confirmed by the results of this study, was based on the HL model adopted in this research and widely disseminated in the literature, where knowledge is an individual ability subdomain. According to this model, familiarity with specific vocabulary and with specific issues related to body functioning and the disease would contribute to HL ^(4)^ . 

Corroborating the findings of this study, the results of a recent survey aimed at characterizing the impact exerted by HL on knowledge and attitudes towards preventive strategies against COVID-19 also did not evidence any association between HL and knowledge (OR = 1.141; 95% CI: 0.981; 1.326, p = 0.086) ^(41)^ . Similarly, a cross-sectional study that evaluated HL and knowledge about diabetes in a sample of 2,895 participants showed no significant associations between HL and knowledge about diabetes (p=0.67) ^(42)^ . 

However, other studies support the association between knowledge about the disease and HL ^(16, 18, 43)^ . A cross-sectional study including 48 patients with atrial fibrillation showed that those with inadequate HL levels had significantly less knowledge about anticoagulant treatment than those with adequate HL levels (55.8 ± 15.9 *vs*. 66.1 ± 14.4, p = 0.02). In addition to that, a lower percentage of patients with inadequate HL levels were aware of the indication for the anticoagulant therapy (57.1% *vs*. 85.2%, p = 0.04), the mechanism of action of the medication (42.9% *vs*. 88.9%, p = 0.001) and its adverse effects (28.6% *vs*. 70.4%, p=0.03) ^(43)^ . 

A meta-analysis of more than 18,000 patients with diabetes showed that higher HL levels were significantly associated with better knowledge about diabetes and, in particular, the performance-based HL measures were the best predictors for knowledge about diabetes ^(18)^ . Interestingly, tools for assessing HL with a numbering section led to a significantly smaller effect size than those that did not include it ^(18)^ . 

In this study, most of the participants answered the numbering questions correctly, which may suggest that the content or the random answer were easy for the sample under study. As a result, the HL score may have been overestimated, affecting the Cronbach’s alpha value. This is not to say that the instrument is not suitable for HL assessments but, rather, that the size of our sample was not sufficient to calculate psychometric parameters. In this sense, it is not possible to draw conclusions regarding validity of the instrument based on our results. Although the sample size was adequate for this purpose, it would be expected that performance of the instrument would not be as good when testing it in people with CAD when compared to the general population, in which it was validated. Studies evaluating the performance of S-TOFHLA in specific groups of people, such as those with CAD, are still required.

This study has limitations that need to be taken into account. The use of convenience sampling from a single center predominantly consisting of white-skinned participants may have compromised heterogeneity of the sample and, therefore, limited generalization of the results. The study did not investigate whether the patients had participated in previous programs that contributed to specific knowledge about the disease or improved their HL-related skills. As this is a cross-sectional research, it was not possible to establish causal relationships. Therefore, our results should be interpreted with caution and further studies should be conducted to confirm our findings.

The findings in this study have relevant implications for the clinical practice. It is important to recognize that hypertensive older adults, with lower schooling levels or unemployed, are more likely to have inadequate HL levels. Therefore, they need to be better supported in their clinical path by someone who can develop and better use the skills in communicating, searching, and processing health information in order to apply them in the daily practice for their own benefit. In this sense, it becomes necessary that health information be transmitted in a clear and objective way, considering the social characteristics that influence HL, as the patients may have difficulty understanding the health information conveyed to them by the team and may often feel uncomfortable to ask for clarifications leading to communication failures and discontinuity of the bond for the care to be provided.

In addition, the health system as a whole should be reorganized to assist patients according to their HL levels to reduce the complexity inherent in navigating across the health services, whether for simple clarification of doubts about their health condition, for scheduling, and for referral to services and other specialized professionals. The interventions need to be focused on helping patients organize the information in a meaningful and simple way so they can put interventions into practice to improve decision-making about their health management.

There is a need for studies on HL levels in different scenarios, as well as for HL assessments to be routinely performed by the health professionals during the first appointment with the patients so that interventions can be designed according to the individual and socioeconomic characteristics and to each patient’s degree of understanding.

## Conclusion

In the scope of this study, HL was influenced by age, hypertension diagnosis, schooling, and professional status but not by specific knowledge about the disease in patients with CAD, suggesting that age, clinical, and social factors may play an important role in the way in which health information is obtained, processed and understood by patients for appropriate decision-making. Health professionals should be aware of the factors that exert an influence on HL, especially in the planning of health interventions, which are more challenging for patients with inadequate HL.
